# ﻿Additions to the genus *Mycena* (Mycenaceae, Agaricales): Descriptions of five new taxa in Hunan Province, China

**DOI:** 10.3897/mycokeys.115.144137

**Published:** 2025-03-28

**Authors:** Ying Xin Xiao, Li Na Liu, Zhu Ming Tan, Ai Rong Shen, Bao Ming Shen, Yun Tan, Sai Nan Li, Li Guo Feng, Jing Bo Long, Zhu Xiang Liu

**Affiliations:** 1 College of Biology and Environmental Sciences, Jishou University, Jishou 416000, China Jishou University Jishou China; 2 Institute of Biodiversity Studies, Hunan Academy of Forestry, Changsha 410004, China Institute of Biodiversity Studies, Hunan Academy of Forestry Changsha China; 3 Hunan Edible Fungi Research Institute, Changsha 410004, China Hunan Edible Fungi Research Institute Changsha China; 4 Huangsang National Nature Reserve Management Office, Shaoyang 422000, China Huangsang National Nature Reserve Management Office Shaoyang China

**Keywords:** Basidiomycota, biodiversity, five new species, phylogenetic analysis, taxonomy

## Abstract

Few studies have been conducted on *Mycena* species in Hunan Province, China. During our research on the species diversity of *Mycena* in Hunan Province, we identified approximately 30 *Mycena* species based on morphological and molecular evidence. Five species are recognized herein as new to science, namely, *M.fulvomarginata*, *M.huangsangensis*, *M.hongfengensis*, *M.subroriduliformis*, and *M.roseolamellata*. The phylogenetic analyses of a combined ITS and LSU sequence dataset revealed that five new species each formed an independent lineage that could separate phenotypically similar and phylogenetically related species. Descriptions, photographs, and phylogenetic analysis results are provided for the five new species, along with the comparisons with related species. A key to all *Mycena* species found in Hunan is also provided.

## ﻿Introduction

Hunan Province is located in the southern middle reaches of the Yangtze River, China, and covers an area of approximately 211,800 km^2^ (Gao and Dou 1981). The province is surrounded by mountains and hills in the east, west, and south, basins in the center, and plains in the north. This area has a subtropical humid monsoon climate with four distinct seasons, sufficient sunshine, and abundant rainfall, which benefits vegetation (Qing 1990). The people’s government of Hunan Province reported that there are 17 national nature reserves, including 16 focused on preserving forest ecological systems (https://www.hunan.gov.cn/). The unique topography and good forest ecological systems make Hunan home to macrofungi (Liu et al. 2024), among which the members of *Mycena* (Pers.) Roussel are prominent in Agaricales (Liu et al. 2022a, 2022b).

*Mycena* is one of the largest genera in the Mycenaceae family of Agaricales, including at least 600 species worldwide (Kirk et al. 2008; Fan et al. 2024; Zhang et al. 2024). *Mycena* plays an important role in ecosystems, which depend on the strong decomposition abilities of fungi to promote material circulation, forest metabolism, and natural renewal (Fukasawa et al. 2009; Guerreiro et al. 2023). Some *Mycena* are symbiotic fungi with *Gastrodiaelata* seeds (Lee et al. 2017; Liu et al. 2022b, 2025). Moreover, approximately 60 *Mycena* species are reported to be bioluminescent worldwide (Chew et al. 2014, 2015; Cortés-Pérez et al. 2019, 2023; Lu et al. 2024).

*Mycena* was first moved to the genus level by Roussel in 1806, but this change was not widely accepted by the majority of mycologists until the 20^th^ century (Roussel 1806). It is a widely studied genus despite many species in the genus having small basidiomata (Aronsen and Læssøe 2016). Many important contributions about taxonomic studies of *Mycena* have been made to temperate regions, and several monographs have been published (Smith 1947; Robich 2003, 2016; Aronsen and Læssøe 2016). For subtropical and tropical areas, the study of local floras has contributed to the early reporting of *Mycena* species (Métrod 1949; Singer and Digilio 1951; Pegler 1977, 1983, 1986, 1987), and those studies made substantial contributions to more important papers and related monographs on *Mycena* (Singer 1989; Corner 1994; Grgurinovic 1998, 2003; Perry 2002; Cooper et al. 2018; Bau et al. 2021).

Some classifications have been proposed based on the morphological characteristics of *Mycena* (Fries 1821; Lange 1914; Kühner 1926, 1931, 1938; Singer and Digilio 1951; Singer 1986; Maas Geesteranus 1992a, 1992b; Maas Geesteranus and Horak 1995; Bau et al. 2021). Currently, mycologists tend to accept, use, and update the infrageneric classification, which was proposed by Maas Geesteranus (Grgurinovic 1998, 2003; Robich 2003, 2016; Cooper et al. 2018). Species are classified into 44 sections based on a combination of macroscopic and microscopic features. With the development of molecular biology research, the phylogenetic positions of *Mycena* have become increasingly apparent (Moncalvo et al. 2002; Matheny et al. 2006; Wei et al. 2024). As a part of the subtropical area of China, studies on *Mycena* have been conducted in Hunan Province. The book Hunan Macrofungi was specially written in 1997 to document the fungi in the region and described two *Mycena* species (Li et al. 1993). Five *Mycena* species from Hupingshan, Hunan, are described in the Atlas of Macrofungi (Zhang et al. 2005). Seven *Mycena* species were recorded in the Atlas of Macrofungi in Hunan (Chen and Zhang 2019). A species in China, *M.heteracantha* (Singer) Desjardin, which was collected from Hunan Province, was described by Na Q and Bau T in 2019 (Na and Bau 2019a). *Mycenasubpiligera* L.N. Liu, a new species collected in Hunan Province that can significantly enhance the germination efficiency of *Gastrodiaelata* seeds, was reported in 2022 (Liu et al. 2022b). *Mycenachlorocyanea* L.N. Liu, another new species whose type was collected in Hunan Province, was reported in recent years (Liu et al. 2022a). As a region that is rich in natural resources, further studies on *Mycena* in Hunan Province are needed.

Aiming to explore the diversity of *Mycena* in the region, we conducted literature reviews and field investigations in Hunan Province from 2020 to 2024. Through macroscopic comparison and phylogenetic analysis, we found 30 *Mycena* species collected from Hunan Province, including five new species that are described in this paper.

## ﻿Materials and methods

### ﻿Sample collection and morphological description

Approximately 400 *Mycena* specimens belonging to 30 taxa were collected from Hunan Province (Table 1). During fieldwork, the collected samples were photographed, and additional information, such as elevation, habitat, and collection date, was recorded (Rathnayaka et al. 2024). Detailed morphological features, including basidiomata size, color and shape, odor, taste, and viscosity, were also documented from fresh specimens. The specimens were dried with silica gel and deposited in the Herbarium of Hunan Institute of Forestry (HUIF) and the Herbarium of Jishou University (JSU). The macromorphological characteristics of the samples were determined based on field notes and photographs. The color codes followed Kornerup & Wanscher (Kornerup and Wanscher 1978). Micromorphological characters of dry specimens were observed under light microscopy (Olympus BX51). To measure the sizes of related tissues, basidiospores, basidia, pileipellis, stipitipellis, and other tissues were mounted in pure water, 5% KOH solution, or 1% Congo Red solution. Melzer’s reagent was used to test the amyloid reaction of the spores. At least 30 basidiospores were measured in each sample. The Q value refers to the length/width ratio of the basidiospores. Q ± av represents the average Q of all basidiospores ± the sample standard deviation. The ranges of the basidiospores are presented as (a–) b–c (–d): the range ‘b–c’ represents 90% of the measured values, while ‘a’ and ‘d’ represent extreme values. The measurements of other microscopic structures were based on 20 measurements per specimen.

**Table 1.** Summary of the collection of *Mycena* species and their distributions and seasons in Hunan.

Species name	Ecology and location (in Hunan)	Occurrence time (in Hunan)	Geographical compositions	References
*М.abramsii*	Solitary, in small groups or subfasciculate on dead twigs or woody debris of deciduous trees; also occurs occasionally on dead twigs of coniferous trees. Widespread in Hunan.	May to July	Worldwide distribution	(Singer and Digilio 1951; Hintikka 1963; Watling 1984; Maas Geesteranus 1988; Emmett 1992; Marra 2000; Tholl et al. 2000; Veerkamp 2001; Laganà et al. 2002; Perry 2002; Robich 2003; Afyon et al. 2005; Bronckers and Kelderman 2011; Senn-Irlet et al. 2012; Gierczyk et al. 2015; Aronsen and Læssøe 2016; Rudolf et al. 2016; Vishwakarma et al. 2017; Bau et al. 2021)
*М.adnexa*	In small groups on dead twigs of deciduous trees. Widespread in Hunan.	May to June	Worldwide distribution	(Bau et al. 2021)
*М.algeriensis*	Subfasciculate on rotten stumps in deciduous forests. Collected only in western areas of Hunan.	April to May	Temperate-subtropical and tropical distribution	(Hintikka 1963; Laganà et al. 2002; Gierczyk et al. 2015; Aronsen and Læssøe 2016; Bau et al. 2021)
*М.bicystidiata*	Scattered on rotten wood in mixed forests. Collected in western and eastern areas of Hunan.	April to June	Only found in China	(Bau et al. 2021)
*М.castaneicola*	Scattered or gregarious on *Castaneamollissima* fruits. Collected only in western areas of Hunan.	June to July	Only found in China	(Bau et al. 2021)
*М.chlorocyanea*	Gregarious in leaf humus under deciduous trees. Collected only in western areas of Hunan.	April to May	Only found in China	(Liu et al. 2022a)
*М.citrinomarginata*	Gregarious in leaf humus under deciduous trees. Collected in western and eastern areas of Hunan.	May to June	Temperate-subtropical and tropical distribution	(Smith 1935; Hintikka 1963; Watling 1984; Senn-Irlet 1987; Emmett 1992; Laganà et al. 2002; Perry 2002; Kalamees and Raitviir 2006; Senn-Irlet et al. 2012; Gierczyk et al. 2015; Gáperová et al. 2015; Na and Bau 2018; Lee et al. 2021)
*М.corynephora*	Gregarious on bark of living deciduous trees. Widely distributed in Hunan.	April to May	Worldwide distribution	(Desjardin 1995; Laganà et al. 2002; Aronsen and Læssøe 2016; Bau et al. 2021)
*М.deeptha*	Gregarious on rotten wood of living deciduous trees. Collected in western and southern areas of Hunan.	April to July	Temperate-subtropical and tropical distribution	(Aravindakshan et al. 2012)
*М.digitifurcata*	Gregarious on bark of living deciduous trees. Collected only in Changsha.	June	Only found in China	(Wei et al. 2024)
*М.filopes*	Solitary on dead twigs of deciduous trees. Collected only in western areas of Hunan.	October	Worldwide distribution	(Beardslee and Coker 1924; Watling 1984; Kalamees and Raitviir 2006; Bronckers and Kelderman 2011; Senn-Irlet et al. 2012; Aronsen and Læssøe 2016; Bau et al. 2021; Cho and Chung 2023)
*М.fulvomarginata*	Gregarious on moss-covered bark of living deciduous trees. Collected only in western areas of Hunan.	June to July	Only found in China	This study
*М.galericulata*	Solitary to fasciculate on branches, trunks and stumps of deciduous trees. Collected only in western areas of Hunan.	October to November	Worldwide distribution	(Beardslee and Coker 1924; Swartz 1933; Imai 1938; Hintikka 1963; Ballesteros 1984; Watling 1984; Maas Geesteranus 1992c; Nauta and Vellinga 1992; Marra 2000; Tholl et al. 2000; Laganà et al. 2002; Perry 2002; Robich 2003; Senn-Irlet et al. 2012; Gáperová et al. 2015; Aronsen and Læssøe 2016; Gyosheva et al. 2016; Vishwakarma et al. 2017; Cho and Chung 2020, 2023; Bau et al. 2021; Lee et al. 2021; Łuszczyński et al. 2022)
*М.haematopus*	In small groups or fasciculate on twigs and trunks of deciduous trees. Distributed in eastern, western and southern areas of Hunan.	May, June, October, November	Worldwide distribution	(Beardslee and Coker 1924; Swartz 1933; Imai 1938; Hintikka 1963; Watling 1984; Emmett 1992; Grgurinovic 1998; Laganà et al. 2002; Perry 2002; Robich 2003; Bronckers and Kelderman 2011; Senn-Irlet et al. 2012; Aravindakshan and Manimohan 2013b; Aronsen and Læssøe 2016; Mustafabayli and Aghayeva 2019; Bau et al. 2021; Cho and Chung 2023)
*М.heteracantha*	Gregarious on decaying leaves and twigs of deciduous trees. Collected only in southwestern areas of Hunan.	May	Temperate-subtropical and tropical distribution	(Desjardin 1995; Bau et al. 2021)
*М.hongfengensis*	Gregarious on decaying leaves of deciduous trees. Collected only in western areas of Hunan.	April	Only found in China	This study
*М.huangsangensis*	Gregarious on decaying leaves of deciduous trees. Collected only in southwestern areas of Hunan.	April to May	Only found in China	This study
*М.laevigata*	In small groups or fasciculate on twigs and trunks of deciduous trees. Collected in western and southern areas of Hunan.	June to September	Temperate-subtropical and tropical distribution	(Chen and Zhang 2019)
*М.leaiana*	Fasciculate on rotten wood of deciduous trees. Collected in western and southern areas of Hunan.	July	Temperate-subtropical and tropical distribution	(Chen and Zhang 2019)
*М.leptocephala*	Gregarious on moss-covered hardwood or on branches. Collected in western and southern areas of Hunan.	April to May	Worldwide distribution	(Beardslee and Coker 1924; Smith 1935; Hintikka 1963; Watling 1984; Senn-Irlet 1987; Laganà et al. 2002; Kalamees and Raitviir 2006; Bronckers and Kelderman 2011; Senn-Irlet et al. 2012; Gierczyk et al. 2015; Aronsen and Læssøe 2016; Baldrian et al. 2016; Bau et al. 2021; Łuszczyński et al. 2022)
*М.maculata*	Solitary to fasciculate on branches, trunks and stumps of deciduous trees. Collected in western areas of Hunan.	October to November	Temperate distribution	(Marra 2000; Tholl et al. 2000; Laganà et al. 2002; Perry 2002; Bau et al. 2021)
*М.meliigena/juniperina*	Gregarious on moss-covered bark of living deciduous trees. Widespread.	November to June	Temperate distribution	(Aronsen 1996; Doğan and Karadelev 2006; Halama et al. 2014)
*М.pearsoniana*	Scattered in leaf humus in deciduous trees. Collected only in western areas of Hunan.	May to June	Worldwide distribution	(Hintikka 1963; Watling 1984; Moreno and Albertó 1996; Robich 2003; Senn-Irlet et al. 2012; Aronsen and Læssøe 2016; Türkekul 2017; Vishwakarma et al. 2017; Kwon et al. 2020; Bau et al. 2021)
*М.picta*	Scattered on decaying leaves of deciduous trees. Collected only in some parks of Changsha.	April to May	Temperate-subtropical and tropical distribution	(Miyamoto et al. 1996; Halama and Romański 2010; Shiryaeva 2018; Retnowati et al. 2020; Bau et al. 2021)
*М.pluteoides*	Solitary or gregarious on rotten wood of deciduous trees. Collected in western and southern areas of Hunan.	May, June	Only found in China	(Bau et al. 2021)
*М.pura*	Scattered in leaf humus and on needles or in grasslands, on both deciduous and coniferous trees. Widely distributed in Hunan.	November, March to June	Worldwide distribution	(Beardslee and Coker 1924; Swartz 1933; Imai 1938; Hintikka 1963; Watling 1984; Senn-Irlet 1987; Emmett 1992; Maas Geesteranus 1992c; Marra 2000; Laganà et al. 2002; Perry 2002; Robich 2003; Kalamees and Raitviir 2006; Senn-Irlet et al. 2012; Casabón 2015; Gáperová et al. 2015; Aronsen and Læssøe 2016; Gyosheva et al. 2016; Mustafabayli and Aghayeva 2019; Bau et al. 2021; Lee et al. 2021)
*М.roseolamellata*	Gregarious on decayed twigs of bamboo or woody debris of deciduous trees. Ningxiang, Hunan.	November, December and March	Only found in China	This study
*М.subpiligera*	Longshan and Suining Counties, Hunan.	April to July	Only found in China	(Liu et al. 2022b)
*М.subroriduliformis*	Gregarious on decaying leaves of deciduous trees. Suining County, Hunan.	April to May	Only found in China	This study
*М.yuezhuoi*	Scattered on the litter layer in *Pinus*, *Quercus*, and *Robinia* mixed forests. Suining County, Hunan.	April to May	Temperate-subtropical and tropical distribution	(Liu et al. 2021; Cho et al. 2024)

### ﻿DNA extraction, PCR amplification, and sequencing

Genomic DNA was extracted from fresh or dried specimens using the NuClean Plant Genomic DNA kit (Kangwei Century Biotechnology Co., Beijing, China) following the manufacturer’s protocols. The ITS rDNA region (ITS1–5.8S–ITS2) was amplified using the primer pair ITS1 and ITS4 (White et al. 1990). The LSU region was amplified with the primers LR0R and LR7 (Vilgalys and Hester 1990). PCR was performed in a total volume of 25 μL containing 1 μL template DNA, 9.5 μL distilled water, 1 μL of each primer, and 12.5 μL 2x Taq PCR Master Mix with blue dye (Sangon Biotech, Shanghai, China). The PCR conditions were as follows: initial denaturation at 95 °C for 5 min, followed by 35 cycles of 94 °C for 45 s, 45 s at 52 °C, and 1 min at 72 °C for ITS (Liu et al. 2022a). For the LSU conditions: initial denaturation at 95 °C for 4 min, followed by 35 cycles of 95 °C for 1 min, 1 min at 53 °C, and 80 s at 72 °C (Zhang et al. 2024). The amplified products were determined by electrophoresis on a 1% agarose gel against a known standard DNA marker and directly sequenced at Sangon Biotech. Newly generated sequences in this study have been submitted to the NCBI GenBank database.

### ﻿Molecular phylogeny

Details of the sequences used for phylogenetic analysis were obtained from this study and downloaded from GenBank (Table 2). DNA sequences were checked using Bioedit v7.0.9 to ensure sequencing quality (Hall 1999). SeqMan 7.1.0 was used for splicing and manual editing (Swindell and Plasterer 1997). The final datasets were aligned using MAFFT v.7.310 (Katoh and Standley 2013). The sequences were concatenated into one multi-loci dataset with SequenceMatrix 1.7.8 (Vaidya et al. 2011). The ALTER (Alignment Transformation EnviRonment) online tool was used for the final conversion of the FASTA format to the NEXUS format (Glez-Penñ et al. 2010). The best-fit evolutionary model was selected using MrModelTest v.2.3 under the Akaike information criterion (AIC) (Nylander 2004). A phylogenetic tree was constructed based on maximum likelihood and Bayesian inference methods. Maximum likelihood (ML) analyses were performed with RAxML-NG v.0.9.0 (Kozlov et al. 2019), and bootstrap values were calculated from 1,000 replicates. Bayesian inference analysis was performed using the Metropolis-coupled Markov chain Monte Carlo method with MrBayes v3.2.5 under the GTR +I+G model (Ronquist and Huelsenbeck 2003). Analyses were run with 4 chains of 2,000,000 generations, and trees were sampled every 100^th^ generation. The first 25% of the sample trees were discarded as burn-in. Gaps were treated as missing data. Phylogenetic trees were visualized with FigTree v1.4.3 (http://tree.bio.ed.ac.uk/software/figtree/).

**Table 2.** Names, voucher numbers, locations, and corresponding GenBank accession numbers of the taxa used in the phylogenetic analysis. - refers to the data unavailability.

Species	Voucher	GenBank accession no.		Location	References
		ITS	LSU		
*Atheniellaadonis*	H6036863	MW540691	-	Finland	Unpublished
*A.aurantiidisca*	UBCF33062	MF908459	-	Canada	Unpublished
*Clitocybulaintervenosa*	BAP 588	MH414560	-	Africa	(Cooper et al. 2018)
*C.intervenosa*	BAP 613	MH414561	MH385335	Africa	(Cooper et al. 2018)
*Hydropusmurinus*	BAP 657	MH414565	-	Africa	(Cooper et al. 2018)
*Mycenaabramsii*	HUIFS50116	OP604436	OP605596	China	Unpublished
*M.abramsii*	HUIFS50074	OP604427	-	China	Unpublished
*M.abramsii*	HUIF50533	PQ406957	-	China	This study
*M.adnexa*	HMAJU43360	MK733290	MK722345	China	Unpublished
*M.adnexa*	HMAJU43691	MK733293	MK722346	China	Unpublished
*M.adnexa*	HUIF50339	PQ406958	-	China	This study
*M.adnexa*	HUIF60005	PQ465300	-	China	This study
*M.albiceps*	F27622	MZ303026	-	USA	Unpublished
*M.albiceps*	RA705-6	MK234177	-	USA	Unpublished
*M.algeriensis*	HMAS 291753	OR236986	-	China	Unpublished
*M.algeriensis*	HUIF50368	PQ406959	-	China	This study
*M.alniphila*	904	JF908482	-	Italy	Unpublished
*M.amicta*	CBS:254.53	MH857183	-	France	(Vu et al. 2019)
*M.amicta*	CBS:352.50	MH856655	MH868170	France	(Vu et al. 2019)
*M.arcangeliana*	252b	JF908401	-	Spain	(Osmundson et al. 2013)
*M.arcangeliana*	252f	JF908402	-	Spain	(Osmundson et al. 2013)
*M.bicystidiata*	HMJAU43648	MK309773	MK629359	China	(Na and Bau 2019b)
*M.bicystidiata*	HUIF50044	PQ406952	-	China	This study
*M.bicystidiata*	HUIF50583	PQ406953	-	China	This study
*M.breviseta*	BAP 633	MH414551	MH385327	Africa	(Cooper et al. 2018)
*M.brunnescens*	JSU125	ON778578	OP360941	China	(Zhang et al. 2024)
*M.brunnescens*	JSU126	ON778579	OP360942	China	(Zhang et al. 2024)
*M.brunnescens*	JSU127	PP152232	-	China	(Zhang et al. 2024)
*M.bulliformis*	SFSU:BAP 547	KX513844	KX513848	USA	(Perry and Desjardin 2016)
*M.caeruleomarginata*	FFAAS0358	OL711670	OL711665	China	(Na et al. 2022)
*M.caeruleomarginata*	FFAAS0357	OL711669	OL711664	China	(Na et al. 2022)
*M.capillaripes*	HRL2854	PQ811198	-	USA	Unpublished
*M.castaneicola*	JSU138	PQ406949	-	China	This study
*M.castaneicola*	JSU263	PQ406950	-	China	This study
*M.castaneicola*	HMJAU43581	MH136827	-	China	(Na and Bau 2019a)
*M.cf.cinerella*	173	MF926553	-	Russia	(Malysheva et al. 2017)
*M.chlorocyanea*	HUIF50234	OP358280	OP360937	China	(Liu et al. 2022a)
*M.chlorocyanea*	HUIF50238	OP358281	OP360938	China	(Liu et al. 2022a)
*M.chlorophos*	CT15101401	MH400938	-	China	Unpublished
*M.cinerella*	Aronsen051014	KT900146	-	Norway	Unpublished
*M.citrinomarginata*	SHXG	OM228755	OM228763	China	Unpublished
*M.citrinomarginata*	HMJAU 43563	MG654739	-	China	(Na and Bau 2018)
*M.confinationis*	MO362993	PP831662	-	USA	Unpublished
*M.confinationis*	PAMP-fungi-41	MT764847	MT764850	Spain	Unpublished
*M.corynephora*	JSU145	PQ406951	-	China	This study
*M.corynephora*	SJiao	OP604434	-	China	Unpublished
*M.cristinae*	JS347	MT921381	MT921384	Brazil	(Oliveira et al. 2021)
*M.cristinae*	JS767	MT921382	-	Brazil	(Oliveira et al. 2021)
*M.crocea*	S.D. Russell iNaturalist #16588497	OM473679	-	USA	Unpublished
*M.crocea*	OMDL K. Canan iNaturalist 182892200	PP436589	-	USA	Unpublished
*M.cyanorhiza*	J24082010	MW540696	-	Finland	Unpublished
*M.cyanorhiza*	120b	JF908385	-	Italy	(Osmundson et al. 2013)
*M.deeptha*	DM334g (K(M)178333)	JX481737	-	India	(Aravindakshan et al. 2012)
*M.deeptha*	HUIF50518	PQ406962	-	China	This study
*M.digitifurcata*	HUIF60006	PQ406940	-	China	(Wei et al. 2024)
*M.digitifurcata*	FFAAS1055	PP706100	PP704700	China	(Wei et al. 2024)
*M.entolomoides*	HMJAU 43126	MG654738	-	China	(Na and Bau 2018)
*M.entolomoides*	HMJAU 43052	MG654737	-	China	(Na and Bau 2018)
*M.entolomoides*	HMJAU 43048	MG654736	-	China	(Na and Bau 2018)
*M.filopes*	HUIF50198	OP604441	OP605599	China	Unpublished
*M.filopes*	HMAS 291835	OR236988	-	China	Unpublished
*M.flosoides*	HUIF50128	OP358282	OP360939	China	(Liu et al. 2022a)
*M.flosoides*	HUIF50129	OP358283	OP360940	China	(Liu et al. 2022a)
*M.flosoides*	HUIF50128-R	OP745013	-	China	(Liu et al. 2022a)
*M.fulgoris*	ACP1690	MG926694	-	Mexico	(Cortés-Pérez et al. 2019)
*M.fulgoris*	ACP1785	MG926693	-	Mexico	(Cortés-Pérez et al. 2019)
***M.fulvomarginata***	**HUIF50088 Holotype**	**PQ406943**	-	**China**	**This study**
***M.fulvomarginata***	**HUIF50089**	**PQ406944**	**PQ406964**	**China**	**This study**
*M.galericulata*	TFB14675	MN088380	-	USA	(Hughes et al. 2020)
*M.galericulata*	TFB14649	MN088382	-	USA	(Hughes et al. 2020)
*M.galericulata*	HUIF50196	OP604439	-	China	Unpublished
*M.haematopus*	HUIF50203	OP604443	OP605601	China	Unpublished
*M.haematopus*	HMJAU43662	MK733299	MK722353	China	Unpublished
***M.huangsangensis***	**HUIF50526 Holotype**	**PQ406935**	-	**China**	**This study**
***M.huangsangensis***	**HUIF50528**	**PQ406936**	**PQ406965**	**China**	**This study**
*M.interrupta*	HMJAU43849	MK733301	-	China	Unpublished
*M.interrupta*	HMJAU43791	MK733300	-	China	Unpublished
*M.juniperina*	869	JF908478	-	Italy	(Osmundson et al. 2013)
*M.laevigata*	HMJAU43618	MK733304	MK722355	China	Unpublished
*M.laevigata*	MHHNU 8626	MK453048	-	China	Unpublished
*M.leaiana*	MHHNU 30544	MK250916	-	China	Unpublished
*M.leaiana*	HKAS126400	OQ025147		China	Unpublished
*M.leptocephala*	HUIF50005	PQ406956	-	China	This study
*M.leptocephala*	CA FUNDIS iNaturalist #160824125	OR778420	-	USA	Unpublished
*M.longinqua*	BAP 648	MH414552	MH385328	Africa	(Cooper et al. 2018)
*M.maculata*	HUIFS50209	OP604446	-	China	Unpublished
*M.maculata*	HMJAU43009	MK309791	MK629347	China	Unpublished
*M.meliigena*	39c	JF908428	-	Italy	(Osmundson et al. 2013)
*M.meliigena*	39	JF908423	-	Italy	(Osmundson et al. 2013)
*M.meliigena*/ *juniperina*	HUIF60003	PQ406954	-	China	This study
*M.meliigena*/ *juniperina*	HUIF60004	PQ406955	-	China	This study
*M.metata*	313b	JF908412	-	Italy	(Osmundson et al. 2013)
*M.metata*	HMJAU43625	MH396636	-	China	Unpublished
***M.hongfengensis***	**JSU114 Holotype**	**PQ406945**	**PQ406967**	**China**	**This study**
***M.hongfengensis***	**JSU121**	**PQ406946**	**PQ406968**	**China**	**This study**
*M.oryzifluens*	FFAAS1051	PP706096	PP704696	China	(Wei et al. 2024)
*M.pasvikensis*	AAronsen50-13	KU861558	-	Norway	Unpublished
*M.pasvikensis*	AAronsen86-12	KU861556	-	Norway	Unpublished
*M.pearsoniana*	TENN61384	JN182200	-	USA	(Harder et al. 2012)
*M.pearsoniana*	TENN61544	JN182199	-	USA	(Harder et al. 2012)
*M.pearsoniana*	HUIF50392	PQ406948	-	China	This study
*M.picta*	CA FUNDIS iNaturalist 171114596	OR858681	-	USA	Unpublished
*M.picta*	TUR194167	MW540717	-	Finland	Unpublished
*M.pluteoides*	HMJAU43771	MK733307	MK722357	China	Unpublished
*M.pluteoides*	HMJAU43765	MK733306	-	China	Unpublished
*M.pluteoides*	HUIF50584	PQ406961	-	China	This study
*M.pluteoides*	HUIF50591	PQ406960	-	China	This study
*M.polygramma*	CBS:240.47	MH856235	MH867764	France	(Vu et al. 2019)
*M.polygramma*	439b	JF908433	-	Italy	(Osmundson et al. 2013)
*M.pura*	HUIF50006	OP604419	OP605597	China	Unpublished
*M.pura*	TENN60139	EU517505	-	Russia	(Petersen et al. 2008)
*M.purpureofusca*	HMJAU 43554	MG654740	-	China	(Na and Bau 2018)
*M.purpureofusca*	HMJAU 43624	MG654741	-	China	(Na and Bau 2018)
*M.rosella*	73h	JF908471	-	Italy	(Osmundson et al. 2013)
*M.rosella*	53	MW576937	-	Norway	Unpublished
***M.roseolamellata***	**HUIF60001 Holotype**	**PQ406941**	**PQ406969**	**China**	**This study**
***M.roseolamellata***	**HUIF60002**	**PQ406942**	-	**China**	**This study**
*M.rubromarginata*	CBS:265.48	MH856335	MH867890	France	(Vu et al. 2019)
*M.rubromarginata*	CBS:268.48	MH856338	MH867891	France	(Vu et al. 2019)
*M.sanguinolenta*	TENN59879	FJ596764	-	USA	(Hughes et al. 2009)
*M.seynesii*	71h	JF908470	-	Italy	(Osmundson et al. 2013)
*M.seynesii*	71I	JF908469	-	Italy	(Osmundson et al. 2013)
*Mycena* sp.	JSU008	PQ465299	-	China	Unpublished
*Mycena* sp.	JSU132	PQ406963	-	China	This stufy
*Mycena* sp.	080108	LC504829	-	Japan	This study
*M.silvaenigrae*	HMJAU43815	MK733310	MK722359	China	Unpublished
*M.subcaerulea*	TENN-F-051121	OL711671	OL711666	USA	(Na et al. 2022)
*M.subcaerulea*	TENN-F-057919	OL711672	OL711667	USA	(Na et al. 2022)
*M.subpiligera*	HUIF50036	OM228758	-	China	(Liu et al. 2022b)
*M.subpiligera*	HUIFS50007	OM228759	-	China	(Liu et al. 2022b)
***M.subroriduliformis***	**HUIF50540 Holotype**	**PQ406937**	**PQ406970**	**China**	**This study**
***M.subroriduliformis***	**HUIF50546**	**PQ406938**	-	**China**	**This study**
*M.substylobates*	HMJAU43444	MH216190	-	China	(Na and Bau 2019a)
*M.substylobates*	HMJAU43418	MH216189	-	China	(Na and Bau 2019a)
*M.tenax*	OSC 113746	EU846251	-	USA	Unpublished
*M.tenax*	OSC 113728	EU669224	-	USA	Unpublished
*M.vulgaris*	447h	JF908435	-	Italy	Unpublished
*M.vulgaris*	CBS:248.47	MH856240	MH867770	France	(Vu et al. 2019)
*M.xantholeuca*	CBS370.50	MH856663	MH868180	France	(Vu et al. 2019)
*M.xantholeuca*	CBS371.50	MH856664	MH868181	France	(Vu et al. 2019)
*M.yuezhuoi*	FFAAS0346	MW581492	-	China	(Liu et al. 2021a)
*M.yuezhuoi*	HUIF50535	PQ406947	-	China	(Liu et al. 2021)
*M.zephirus*	CBS:270.48	MH856339	MH867892	France	(Liu et al. 2021)
*M.zephirus*	AH60146	PP868143	-	Spain	(Villarreal et al. 2024)
*Phloeomanaminutula*	H6036841	MW540684	-	Finland	Unpublished
*P.speirea*	iNAT: 100003394	ON206666	-	USA	Unpublished

## ﻿Results

### ﻿Phylogenetic analysis

**Figure 1.**
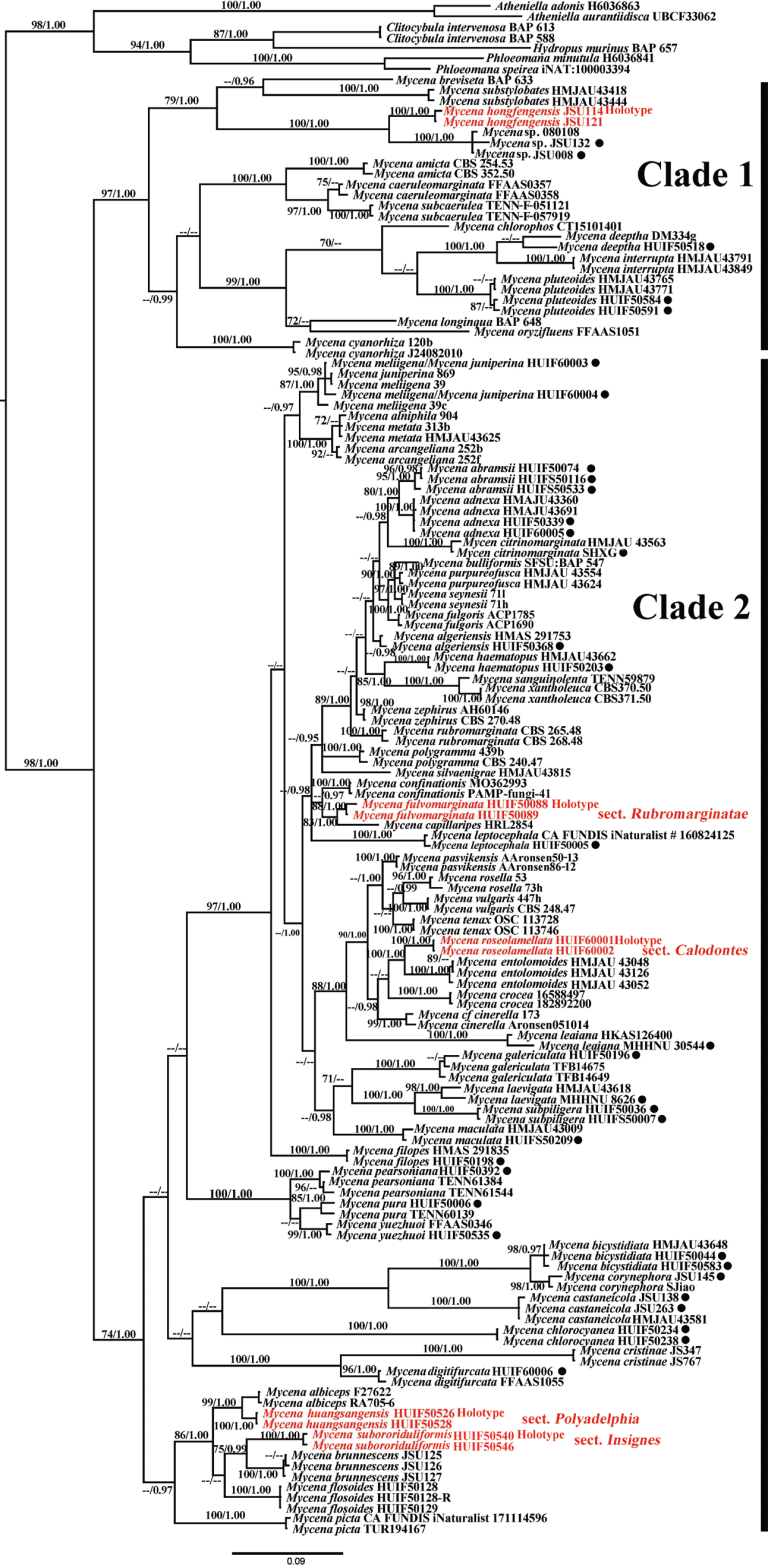
Phylogenetic relationships of *Mycena* species in Hunan Province inferred from the combined dataset (ITS and nrLSU) using Bayesian posterior probabilities (BP) ≥ 0.95; Bootstrap support (BS) ≥ 70% are reported on the branches. Red text represents new taxa. The black dots indicate the *Mycena* species collected from Hunan Province.

The two-locus dataset (ITS + LSU) consisted of 191 sequences and 1,680 nucleotide sites in total, which are shown in Table 2. It includes sequences of 28 *Mycena* taxa except for *M.picta* (Fr.) Harmaja and *M.heteracantha*, which are present in Hunan. Sequences of closely related species with high homology and morphologically similar species were also downloaded from GenBank. *Atheniellaadonis* (Bull.) Redhead, Moncalvo, Vilgalys, Desjardin, & B.A. Perry, *A.aurantiidisca* (Murrill) Redhead, Moncalvo, Vilgalys, Desjardin, & B.A. Perry; *Clitocybulaintervenosa* A.C. Cooper, Desjardin, & B.A. Perry (BAP 588, BAP 613), *Phloeomanaminutula* (Sacc.) Redhead; *P.speirea* (Fr.) Redhead, and *Hydropusmurinus* A.C. Cooper, Desjardin, & B.A. Perry were chosen as the outgroup (Liu et al. 2022a). The topologies generated from maximum likelihood (ML) and Bayesian inference (BI) analyses were identical, although statistical support for some branches showed slight differences. The BI tree with branch lengths inferred from the ITS and LSU datasets is shown in Fig. 1.

The phylogeny inferred from the combined dataset revealed that the *Mycena* split into two well-supported clades, and all new taxa formed a well-supported monophyletic lineage. *Mycenahongfengensis* formed a small branch and grouped with an unidentified *Mycena* sp. in clade 1 (BS/BP = 100/1.00). *Mycenaroseolamellata*, *M.fulvomarginata*, *M.huangsangensis*, and *M.subroriduliformis* were members of clade 2. *Mycenaroseolamellata* and *M.entolomoides* T. Bau formed a supported branch in the tree (BS/BP = 100/1.00), and their genetic distance is substantial enough to distinguish between the two species. *Mycenafulvomarginata* is most related to *M.capillaripes* Peck. They grouped together with BS/BP = 83/1.00 statistical support, and they were distinct. *Mycenaalbiceps* (Peck) Gilliam, *M.flosoide* L.N. Liu, *M.brunnescens* L.N. Liu, and our specimens (*M.huangsangensis* and *M.subroriduliformis*) formed a separate branch with strong statistical support (BS/BP = 86/1.00).

### ﻿Taxonomy

#### ﻿ Mycena huangsangensis

Taxon classificationFungiAgaricalesMycenaceae

L.N. Liu sp. nov.

8A3EBB1D-C524-5FF6-A5CF-51298A3561DB

856016

Figs 2 , 3

##### Diagnosis.

Differs from the most similar species, *M.alniphila*, by its decurrent lamellae and longer basidiospores.

##### Holotype.

China • Hunan Province, Shaoyang City, Suining County, Hunan Huangsang National Nature Reserve, 26°24'18"N, 110°05'37"E, elev. 644 m, 24 April 2024, LiNa Liu, *HUIF50526* (collection number NN526).

##### Etymology.

Refers to the Huangsang National Nature Reserve, from where the holotype was collected.

##### Description.

Pileus 1–5 mm diam., hemispherical to obtusely conical, expanding with age, umbilicate or depressed center, sulcate, translucent-striate, pruinose and pubescent, light brown (6B4–6B7) to dark brown (6E7–6E8), or brownish-pink (7A4–7B4), paler brown towards margin. Context white, thin, fragile. Lamellae 9–11 reach the stipe, with 1–2 tiers of lamellulae, decurrent, white (4A1) to brown (7D7), edge concolorous with face. Stipe 6–25 × 1–2 mm, cylindrical, hollow, fragile, light brown (6B4–6B7) to brown (6E5–6E8) at the base, gradually becoming paler to white (4A1) towards the apex. Base covered with white fibrils. Odor and taste indistinctive.

**Figure 2.**
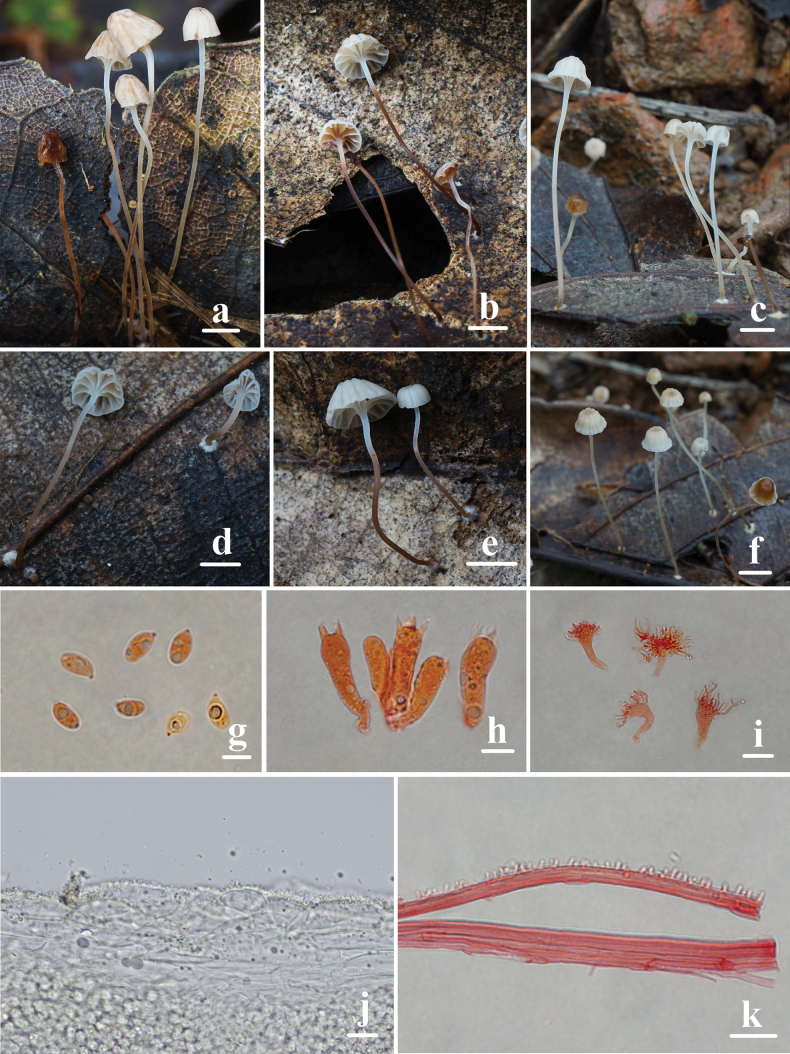
Basidiomata and microscopic features of *Mycenahuangsangensis***a–f** basidiomata **g** basidiospores **h** basidia **i** cheilocystidia **j** pileipellis **k** stipitipellis. Structures (**g–i, k**) were stained in 1% Congo red solution and **j** were rehydrated in 5% KOH solution. Scale bars: 5 mm (**a–f**); 5 μm (**g**); 10 μm (**h–k**).

Basidiospores 6.0–7.4 (7.8) × (3.2) 3.4–4.2 (4.3) μm, Q = 1.4–2.1, Q = 1.8 ± 0.2, pip-shaped, elongated, hyaline, smooth, thin-walled, amyloid. Basidia 15.4–20.9 × 6.2–9.0 μm, 4-spored, clavate. Cheilocystidia 13.2–25.0 × 8.5–19.9 μm, abundant, clavate to obpyriform, covered with fairly numerous, simple to furcate, cylindrical excrescences. 1.2–9.5 × 0.5–1.4 μm. Pleurocystidia absent. Hyphae of the pileipellis 12–27 μm wide, densely covered with warts or short cylindrical excrescences. Hyphae of the stipitipellis 1.0–3.0 μm wide, densely covered with simple, cylindrical excrescences 1.0–3.2 × 0.8–1.5 μm. Clamp connections are present in the basidia, pileipellis, and stipitipellis hyphae.

**Figure 3.**
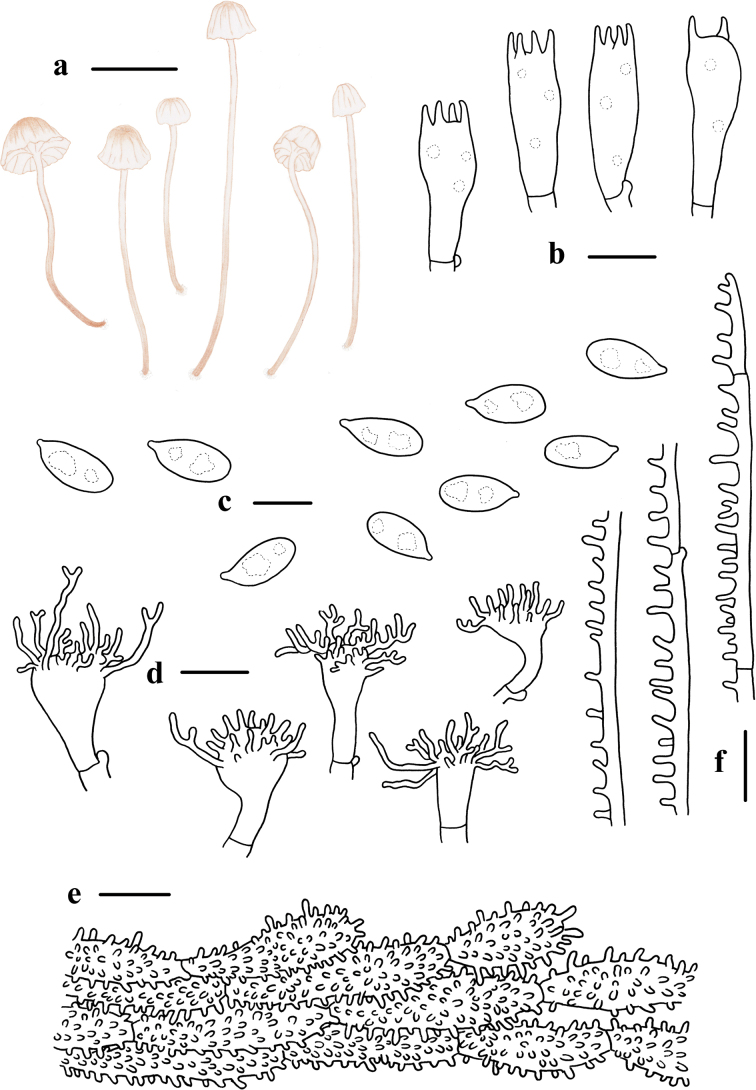
Morphological features of *Mycenahuangsangensis***a** basidiomata **b** basidia **c** basidiospores **d** cheilocystidia **e** pileipellis **f** hyphae of stipitipellis. Scale bars: 1 cm (**a**); 5 μm (**c**); 10 μm (**b, d–f**).

##### Habitat.

Gregarious on decaying leaves of deciduous trees.

##### Known distribution.

Shaoyang City, Hunan Province.

##### Additional materials examined.

China• Hunan Province, Shaoyang City, Suining County, Hunan Huangsang National Nature Reserve, 26°24'21"N, 110°05'36"E, elev. 675 m, 24 April 2024, LiNa Liu, *HUIF50528* (collection number NN528).

##### Notes.

*Mycenahuangsangensis* can be considered to be a member of sect. Polyadelphia owing to very small basidiomata, a small number of lamellae, and a slender stipe and hyphae of the pileipellis, which are ornamented with short warts. *Mycenahuangsangensis* belongs to the section with a brownish pileus, while *M.alniphila* Robich shows the most significant morphological similarity to *M.huangsangensis*. They have similar basidiomata color and shape of cheilocystidia, pileipellis hyphae densely covered with cylindrical excrescences, and diverticulate stipitipellis hyphae. However, *M.alniphila* differs in having adnate lamellae, slightly longer spores measuring 8.5–11.0 × (3.5) 4.0–5.5 μm, simple cheilocystidia without branching, and caulocystidia present (Robich 2003). *Mycenaalbiceps* and *M.catalaunica* Robich are somewhat similar to the new species; in particular, they share the same basidiomata shape and similar habitats. *Mycenaalbiceps* differs in the white colors of the pileus and black stipe (Gilliam 1976; Maas Geesteranus 1986). The latter, *M.catalaunica*, has a pale violaceous pink to pale vinaceous pink pileus, subglobose spores, and caulocystidia; the clamp connection is absent in all tissues, and cheilocystidia are subglobose (Robich 2003).

#### ﻿ Mycena fulvomarginata

Taxon classificationFungiAgaricalesMycenaceae

L.N. Liu sp. nov.

7DAF3517-1E75-583A-ACF3-829A9CE36650

856027

Figs 4 , 5

##### Diagnosis.

Differs from the closest species, *M.rubromarginata*, in having yellow lamellae edges and light-yellow contents in cheilocystidia, hyphae of the pileipellis, and stipitipellis.

##### Holotype.

China• Hunan Province, Suining County, Hunan Huangsang National Nature Reserve, Shaoyang City, 26°25'41"N, 110°03'27"E, elev. 1075 m, 26 June 2021, LiNa Liu, *HUIF50088* (collection number NN88).

##### Etymology.

Refers to the yellow color of the lamellae edges.

##### Description.

Pileus 4–8 mm diam., hemispherical when young, paraboloid or campanulate with age, sulcate, pellucid-striate, pruinose, apex with obtuse umbo, the margin infrequently out of flatness, dark reddish brown (7C8) at center, gradually becoming paler towards the margin to light brown (7A6), turning purple (12B5) with age. Context white, thin, fragile. Lamellae 10–12 reach the stipe, with 1 tier of lamellulae, adnexed, white (4A1), edge yellow (5B7), stipe 6.0–12.0 × 0.5–1.0 mm, central, cylindrical, hollow, fragile, finely white pruinose and pubescent, pale brown (6D7) to brown (6F7), fading to purple (12B5). Base slightly bulbous, covered with white fibrils. Odor and taste indistinctive.

**Figure 4.**
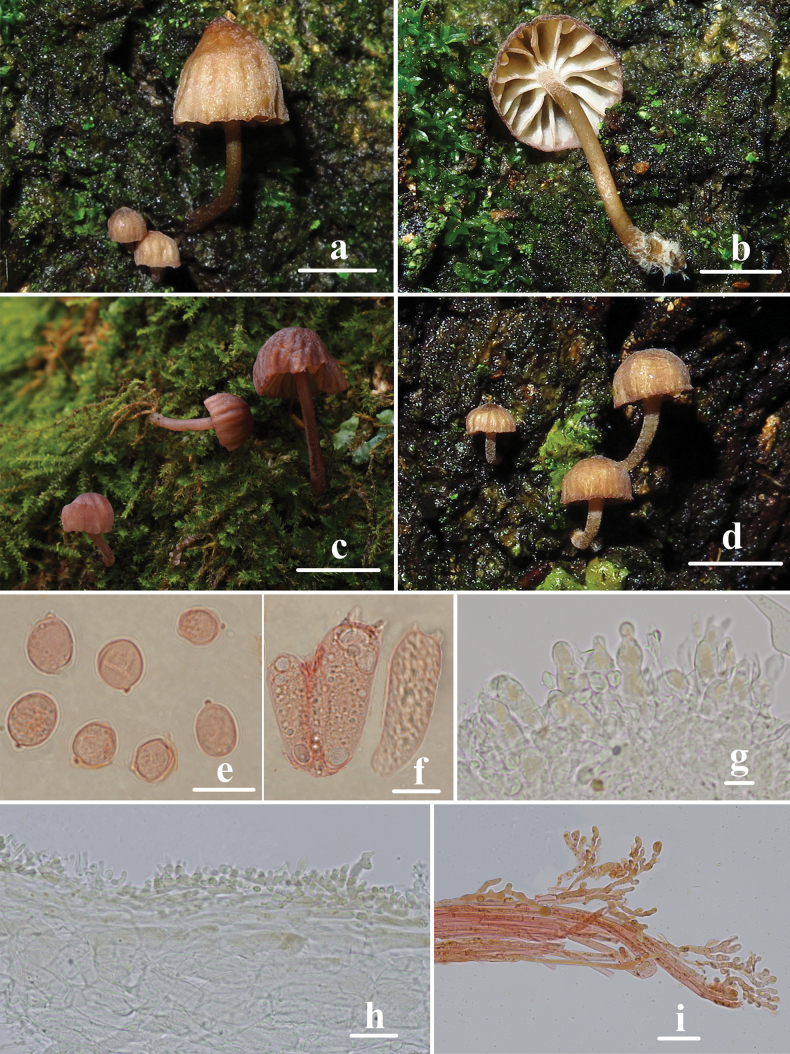
Basidiomata and microscopic features of *Mycenafulvomarginata***a–d** basidiomata **e** basidiospores **f** basidia and basidioles **g** cheilocystidia **h** pileipellis **i** stipitipellis. Structures (**e, f, i**) were stained in a 1% Congo red solution, and **g, h** were rehydrated in a 5% KOH solution. Scale bars: 5 mm (**a–d**); 10 μm (**e–i**).

Basidiospores (7.8) 7.9–9.9 (10.0) × (4.5) 5.7–8.0 (8.2) μm, Q = 1.2–1.7, Q = 1.3 ± 0.1, subglobose to broadly ellipsoid, hyaline, smooth, thin-walled, amyloid. Basidia 24.8–33.5 × 8.2–13.5 μm, 4-spored, clavate. Cheilocystidia 22.6–45.8 × 7.7–17.8 μm, abundant, fusiform to ventricose, clavate, subcylindrical, or somewhat irregularly shaped, smooth or covered with one or more apical simple to furcate excrescences, with light yellow contents. Pleurocystidia absent. Hyphae of the pileipellis 3.0–9.0 μm wide, covered with simple to much-branched excrescences, 2.0–6.0 × 2.0–4.0 μm, with light yellow contents. Hyphae of the stipitipellis 2.0–5.0 μm wide, covered with numerous simple to furcate cylindrical excrescences, 2.0–11.0 × 2.0–8.0 μm, with light yellow contents. Clamp connections are present in the basidia, pileipellis, and stipitipellis hyphae.

**Figure 5.**
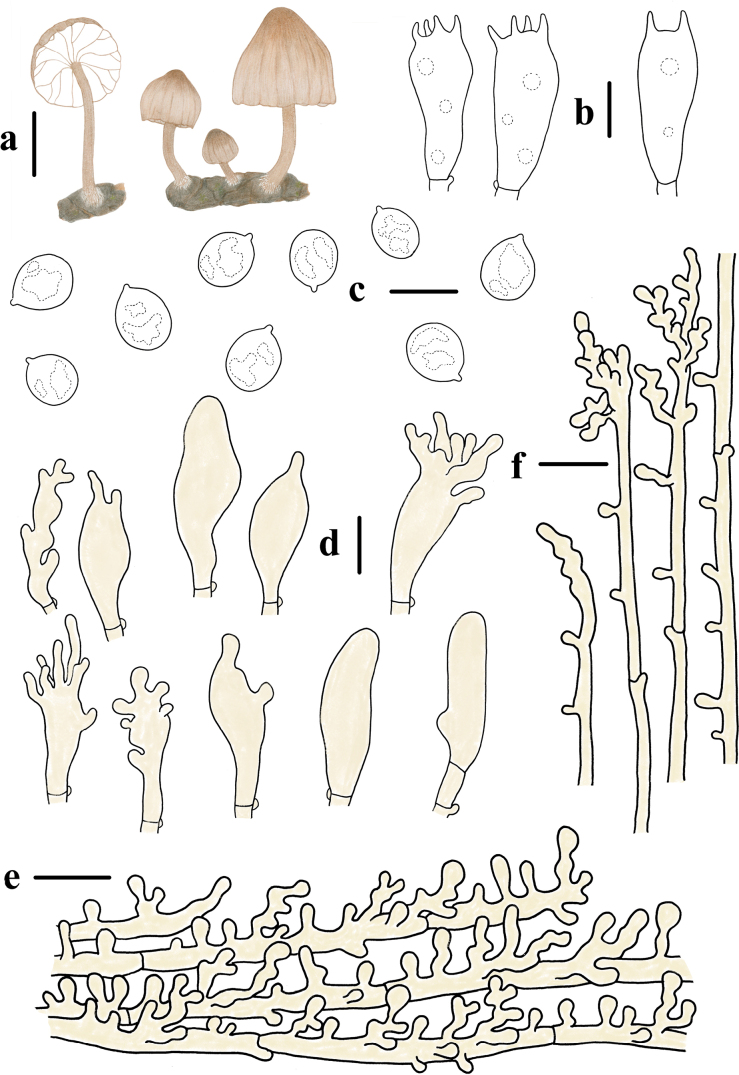
Morphological features of *Mycenafulvomarginata***a** basidiomata **b** basidia **c** basidiospores **d** cheilocystidia **e** pileipellis **f** hyphae of stipitipellis. Scale bars: 5 mm (**a**); 10 μm (**b–f**).

##### Habitat.

Gregarious on moss-covered bark of living deciduous trees.

##### Known distribution.

Hunan Province and Guangxi Zhuang Autonomous Region, China.

##### Additional materials examined.

China • Guangxi Zhuang Autonomous Region, Baise City, Leye County, Yachang Orchid National Nature Reserve, 24°46'29"N, 106°20'09"E, elev. 1080 m, 24 April 2024, LiNa Liu, *HUIF50089* (collection number NN89).

##### Notes.

*Mycenafulvomarginata* belongs to sect. Rubromarginatae Singer ex. Maas Geest. due to the very small basidiomata, yellow lamellar edges, cheilocystidia with colored contents, diverticular pileipellis, and stipitipellis hyphae, along with exhibiting a growth habit on decaying wood. *Mycenafulvomarginata* is similar to the species described in this section: *M.rubromarginata* (Fr.) P. Kumm., *M.seynii* Quél., and *M.bulliformis* B.A. Perry & Desjardin. They resemble *M.fulvomarginata* in the shape of their cheilocystidia, are covered with excrescences on the pileipellis and stipe cortical hyphae, and have a similar brown pileus. However, *M.rubromarginata* differs in that it has longer pileipellis excrescences, up to 36 μm, and cheilocystidia that are 20–65 (up to 90) μm long, with reddish-brown contents (Robich 2003; Aronsen and Læssøe 2016). *Mycenaseynii* should be easy to separate from the new species by its medium basidiomata, larger basidiospores measuring 10.5–15.0 × 6.0–7.5 μm, reddish-purple edge, larger cheilocystidia measuring 30–85 × 8–16 μm, pileipellis, and stipitipellis hyphae with brown to dark red granular contents (Robich 2003; Aronsen and Læssøe 2016). In addition, *M.bulliformis* differs from *M.fulvomarginata* by its violet to violet-brown edges, ellipsoid to broadly ellipsoid spores, and some smooth pileipellis hyphae (Perry and Desjardin 2016).

#### ﻿ Mycena hongfengensis

Taxon classificationFungiAgaricalesMycenaceae

L.N. Liu sp. nov.

14E4D78C-D4A0-59D4-83D8-18071AFBDBD8

856029

Figs 6 , 7

##### Diagnosis.

Differs from *M.castaneicola* in having smooth cheilocystidia, dermatocysts present in the pileipellis, and stipitipellis hyphae.

##### Holotype.

China • Hunan Province, Xiangxi Tujia-Miao Autonomous Prefecture, Jishou City, Hongfeng Forest Park, 28°16'23"N, 109°40'45"E, elev. 230 m, 22 April 2024, ZhuXiang Liu, *JSU114* (collection number JD114).

##### Etymology.

Refers to the Hongfeng Forest Park, from where the holotype was collected.

##### Description.

Pileus 2–5 mm diam., hemispherical when young, becoming nearly campanulate or plano-convex with age, with a centrally flattened depression, margin smooth, sulcate, translucently striate, pure white (4A1), white pubescent. Context pure white, thin, fragile. Lamellae 16–18 reach the stipe, with 1–2 tiers of lamellulae, narrowly free, pure white (4A1), concolorous with the sides. Stipe 15–40 × 0.1–0.5 mm, almost equal or slightly expanding towards the base, hollow, white (4A1) to greyish-white (4B1), pubescent or puberulous, base swollen. With a not well-developed basal disc, covered with white hirsute. Odor and taste not distinctive.

**Figure 6.**
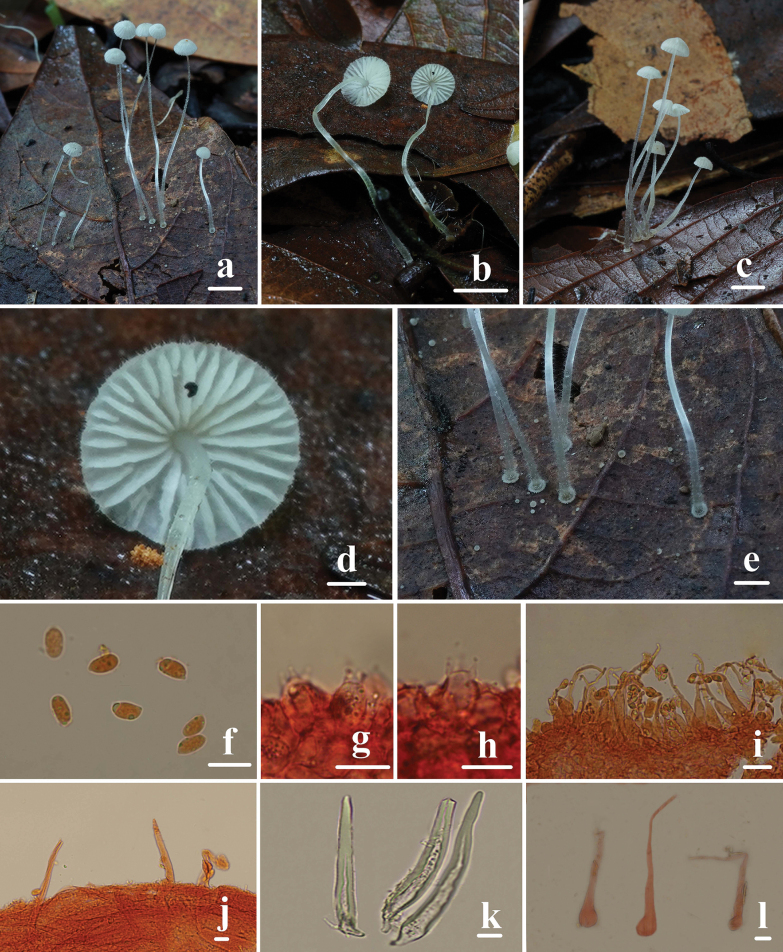
Basidiomata and microscopic features of *Mycenahongfengensis***a–e** basidiomata **f** basidiospores **g, h** basidia **i** cheilocystidia **j, k** dermatocysts in the pileipellis **l** dermatocysts in the stipitipellis. Structures (**f–j, l**) were stained in a 1% Congo red solution and **k** were rehydrated in a 5% KOH solution. Scale bars: 5 mm (**a–c, e**); 1 mm (**d**); 5 μm (**f**); 10 μm (**g–l**).

Basidiospores (6.2) 6.3–7.6 (7.7) × (3.4) 3.5–4.9 (5.2) μm, Q = 1.4–2.0, Qm = 1.7 ± 0.2, oblong or pip-shaped, hyaline, thin-walled, amyloid. Basidia 8–16 × 4–8 μm, two- and four-spored, clavate, hyaline. Cheilocystidia 11–43 × 5–9 μm, obpyriform, fusiform, ventricose, filiform, with a long neck, up to 25 μm, with an acute and occasionally branched apex, hyaline. Pleurocystidia absent. Pileipellis hyphae 2–13 μm wide, hyphae cylindrical, densely covered with warts and cylindrical excrescences, 1.0–6.0 × 1.0–2.0 μm, with irregularly cylindric to strangulated dermatocysts, 63–200 × 7–20 μm, walls 1.0–2.0 μm, greenish grey (1C2). Hyphae of the stipitipellis 1.0–6.0 μm wide, smooth, dermatocysts numerous, clavate to pyriform, 50–320 × 5–20 μm, long, flexuous, filiform, simple, and tapering towards the apex. Clamp connections are absent in the basidia, pileipellis, and stipitipellis hyphae.

**Figure 7.**
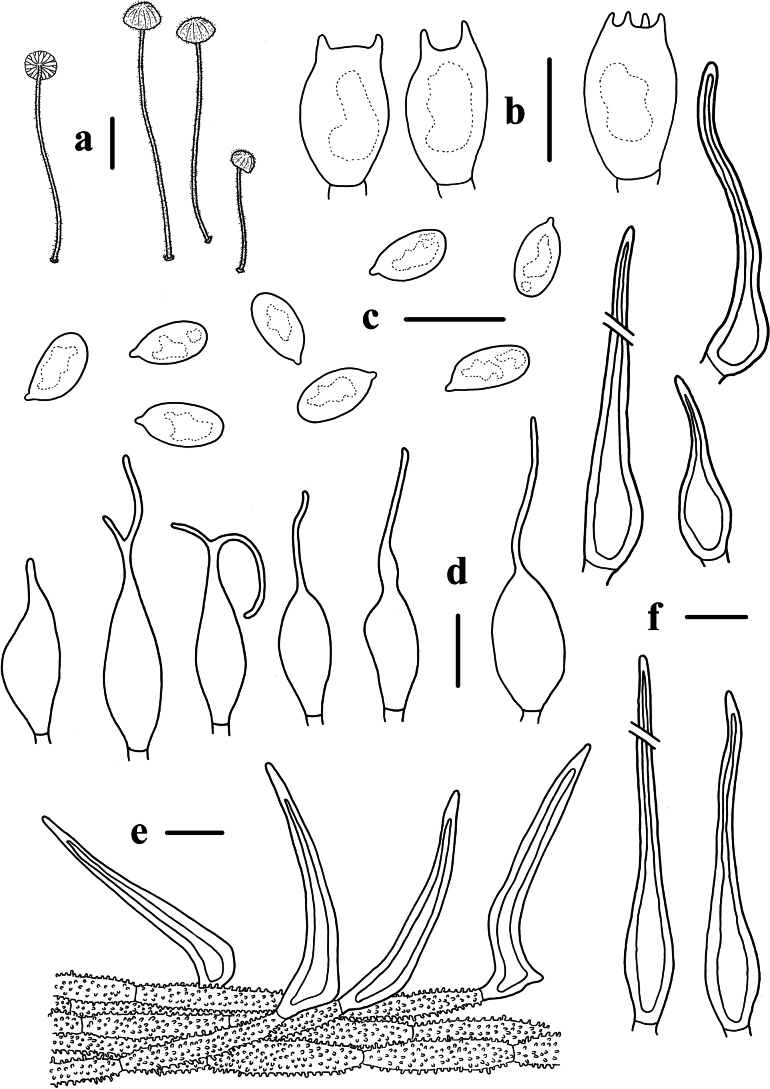
Morphological features of *Mycenahongfengensis***a** basidiomata **b** basidia **c** basidiospores **d** cheilocystidia **e** dermatocysts in the pileipellis **f** dermatocysts in the stipitipellis. Scale bars: 1 cm (**a**); 10 μm (**b–f**).

##### Habitat.

Gregarious on decaying leaves of deciduous trees.

##### Known distribution.

Xiangxi Tujia-Miao Autonomous Prefecture, Hunan Province.

##### Additional materials examined.

China• Hunan Province, Xiangxi Tujia-Miao Autonomous Prefecture, Jishou City, Hongfeng Forest Park, 28°16'26"N, 109°40'48"E, elev. 255 m, 22 April 2024, ZhuXiang Liu, *JSU121* (collection number JD121).

##### Notes.

*Mycenahongfengensis* is characterized by its pure white basidiomata, free lamellae, oblong to pip-shaped spores, and the presence of dermatocysts in the pileipellis and stipitipellis. According to the Maas Geesteranus classification, the new species could belong to an uncertain position. When we first found this specimen in the field, we thought it might be a member of either sect. Saccharifera or sect. Amparoina. All species in two sections have a white basidiomata, pubescent pileus and stipe, and stipe with a basic disc (Maas Geesteranus 1983; Desjardin 1995; Na and Bau 2019b). However, *M.hongfengensis* can be clearly distinguished from other species through microscopic characteristics. The presence of dermatocysts in the pileipellis and stipitipellis is the most important characteristic for separating *M.hongfengensis* from species of sect. Sacchariferae and sect. Amparoina. *Mycenacastaneicola* T. Bau & Q. Na is the most similar to the new species in the macroscopic characteristics, but the microscopic features differ significantly. Sequences labeled as *Mycena* sp., which were extracted from the host *Myricarubra* (Lour.) Siebold & Zucc. and originated from Japan, along with the sequence of *M.hongfengensis*, formed a well-supported lineage in the phylogenetic analysis (BS/BP = 100/1.00). We are very fortunate to have collected *Mycena* sp. in the field. However, we only collected one tiny basidioma; the microscopic structures we observed are not comprehensive. Both taxa have white pileus and pubescent pileus and stipe. The uncertain *Mycena* species differs in pileipellis, which has “acanthocysts” that are spherical or vesicular and covered with spines. These features can be used to easily differentiate the two species.

#### ﻿ Mycena subroriduliformis

Taxon classificationFungiAgaricalesMycenaceae

L.N. Liu sp. nov.

164BC7F6-F4A1-5692-8D36-C9EE068BE02C

856045

Figs 8 , 9

##### Diagnosis.

Differs from *M.surculosa* in having a viscid pileus and stipe.

##### Holotype.

China • Hunan Province, Suining County, Hunan Huangsang National Nature Reserve, Shaoyang City, 26°24'47"N, 110°05'20"E, elev. 542 m, 25 April 2024, LiNa Liu, *HUIF50540* (collection number NN540).

##### Etymology.

Refers to the viscid pileus and stipe of the new species.

##### Description.

Pileus 2–8 mm diam., hemispherical when young, campanulate with age, with obvious depression at the center, sulcate, translucent-striate, surface wet, glabrous, brownish white (7A1), light brown (7C3), brownish grey (7D1–6D2) when old. Context white, thin, and fragile. Lamellae 18–20 reach the stipe, with 1–2 tiers of lamellulae, decurrent, white (4A1), concolorous with the sides. Stipe 2–45 × 1–2 mm, cylindrical, hollow, fragile, surface glutinous, white (4A1) to brownish grey (5A1–5D3) towards the apex, light brown to brown (5D6–6D6) towards the base, base swollen. Odor and taste indistinctive.

**Figure 8.**
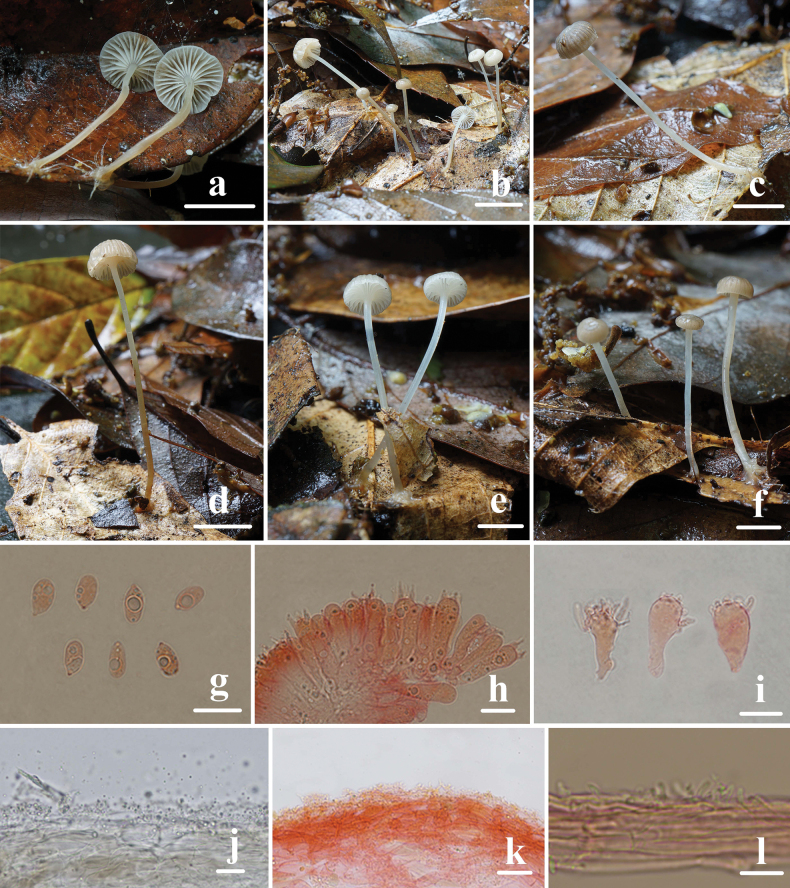
Basidiomata and microscopic features of *Mycenasubroriduliformis***a–f** basidiomata **g** basidiospores **h** basidia **i** cheilocystidia **j, k** pileipellis **l** stipitipellis. Structures (**g–i, k, l**) were stained in a 1% Congo red solution and **j** were rehydrated in a 5% KOH solution. Scale bars: 1 cm (**a–f**); 10 μm (**g–l**).

Basidiospores (6.2) 6.6–8.5 (9.0) × (3.6) 3.8–5.2 (5.3) μm, Q = 1.5–2.1, Q = 1.7 ± 0.1, pip-shaped, cylindrical, hyaline, amyloid, smooth. Basidia 19.3–26.9 × 5.6–8.0 μm, 4-spored, clavate, hyaline. Cheilocystidia 16.8–26.9 × 6.3–17.1 μm, abundant and variably shaped, clavate to cylindrical with short, more or less branched projections, 1.0–6.0 × 1.0–2.0 μm, thin-walled, hyaline. Pleurocystidia absent. Pileipellis hyphae somewhat gelatinized, 2.0–5.0 μm wide, with irregular simple to branched warts or cylindrical excrescences, 1.0–4.0 × 1.0–2.0 μm. Hyphae of the stipitipellis 1.0–6.0 μm wide, covered with cylindrical excrescences. 1.0–4.0 × 1.0–2.0 μm. Clamp connections are present in the basidia, pileipellis, and stipitipellis hyphae.

**Figure 9.**
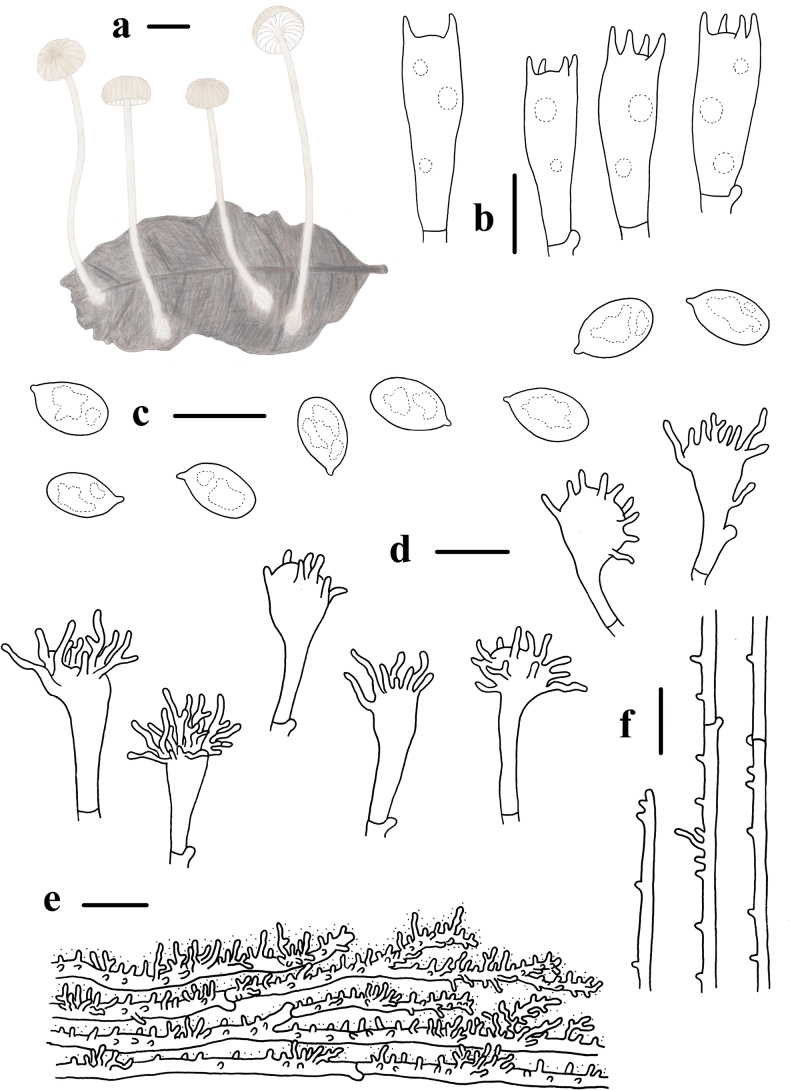
Morphological features of *Mycenasubroriduliformis***a** basidiomata **b** basidia **c** basidiospores **d** cheilocystidia **e** pileipellis **f** hyphae of stipitipellis. Scale bars: 5 mm (**a**); 10 μm (**b–f**).

##### Habitat.

Gregarious on decaying leaves of deciduous trees.

##### Known distribution.

Shaoyang City, Hunan Province.

##### Additional materials examined.

China• Hunan Province, Suining County, Hunan Huangsang National Nature Reserve, Shaoyang City, 26°24'39"N, 110°05'25"E, elev. 588 m, 25 April 2024, LiNa Liu, *HUIF50546* (collection number NN546).

##### Notes.

The following characteristics placed this new species in the sect. Insignes Maas G. due to the viscid pileus and stipe, decurrent lamellae, pip-shaped spores, clavate cheilocystidia with coarse excrescences, hyphae of the pileipellis embedded in gelatinous matter, and diverticulate (Maas Geesteranus 1989). The three other similar species in sect. Insignes are *M.surculosa* Maas G. & de Meijer, *M.odorifera* (Peck) Sacc., and *M.calceata* Robich. All are differentiated from *M.subroriduliformis* as follows: *M.surculosa* has a dry pileus, with only the stipe being viscid. The apical side branches of the pileipellis are densely covered and simple to forked, with cylindrical excrescences. The stipitipellis is somewhat gelatinized (Maas Geesteranus and De Meijer 1997). *Mycenaodorifera* has a distinctive alkaline-like odor, a pruinose stipe, pubescence, and cheilocystidia occasionally with forked apices (Smith 1935). *Mycenacalceata* has dark brown basidiomata and relatively large spores (11.0–13.5 × 5.5–8.0 μm), smooth cheilocystidia, or one or two branches at the apex (Robich 2003).

#### ﻿ Mycena roseolamellata

Taxon classificationFungiAgaricalesMycenaceae

L.N. Liu sp. nov.

E49450B8-55CD-54CA-BE0C-F7E345C98D68

856026

Figs 10 , 11

##### Diagnosis.

Differs from *M.pura* in having a brown pileus and pink lamellae.

##### Holotype.

China• Hunan Province, Ningxiang City, Biandan’ao, Lijingpu Subdistrict, 28°12'07"N, 112°32'43"E, elev. 110 m, 28 November 2023, ShengQiang Liu, *HUIF60001* (collection number NN601).

##### Etymology.

Refers to the pink colors of lamellae.

##### Description.

Pileus 7–17 mm diam., parabolic when young, then campanulate or broadly conical with age, apex with an obtuse umbo, sulcate, translucent-striate, glabrous, dark brown (6F5–6F8) at first, then turning pale brownish yellow (6A4) to pale brown (6E4) with age, margin brownish white (6A2) to pale brown (6D4–6D6). Context white, fragile, thin. Lamellae 24–26 reach the stipe, with 1–2 tiers of lamellulae, adnate or slightly adnex, white (4A1) when young, pinkish to light pink (9A3–9A2) at maturity, concolorous with faces. Stipe 29–30 × 2.0–3.0 mm, central, cylindrical, hollow, dark brown (6F5-6F8), pale brownish yellow (6A4) to pale brown (6D7) with age, base covered with long, dense, white fibrils. Odor and taste indistinctive.

**Figure 10.**
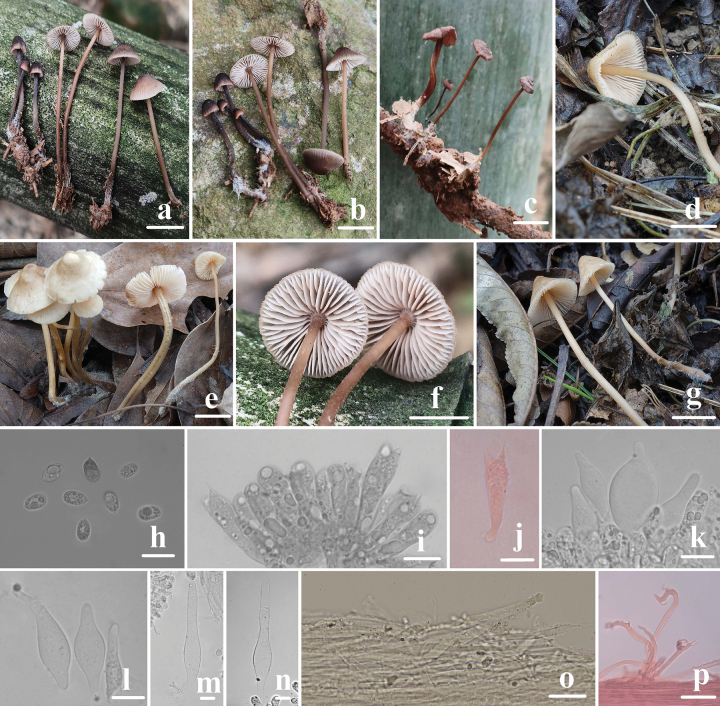
Basidiomata and microscopic features of *Mycenaroseolamellata***a–g** basidiomata **h** basidiospores **i, j** basidia **k, l** cheilocystidia **m, n** pleurocystidia **o** pileipellis **p** stipitipellis. Structures (**j, p**) were stained in 1% Congo red solution, and **h, i, k–o** were rehydrated in 5% KOH solution. Scale bars: 1 cm (**a–g**); 10 μm (**h–p**).

Basidiospores (8.3) 8.6–10.8 (11.5) × (5.3) 5.4–6.3 (6.4) μm, Q = 1.5–2.0, Q = 1.7 ± 0.1, ellipsoid to elongated, hyaline, smooth, thin-walled, amyloid. Basidia 21.7–30.8 × 6.8–8.9 μm, 2-spored, clavate. Cheilocystidia 26.7–84.9 × 8.6–18.7 μm, abundant, fusiform, ventricose-rostrate, obtuse apex, base tapered, with short to long stalk, smooth, hyaline, amyloid, thin-walled. Pleurocystidia similar to cheilocystidia, 52.3–105.7 × 12.3–20.9 μm. Pileipellis 1.0–6.0 μm wide, smooth, terminal hyphae sometimes diverticulate, 1.0–6.0 × 1.0–2.0 μm. Stipitipellis 2.0–5.0 μm, smooth, terminal hyphae sometimes diverticulate, 1.0–4.0 × 1.0–2.0 μm. Clamp connections are absent in the basidia, pileipellis, and stipitipellis hyphae.

**Figure 11.**
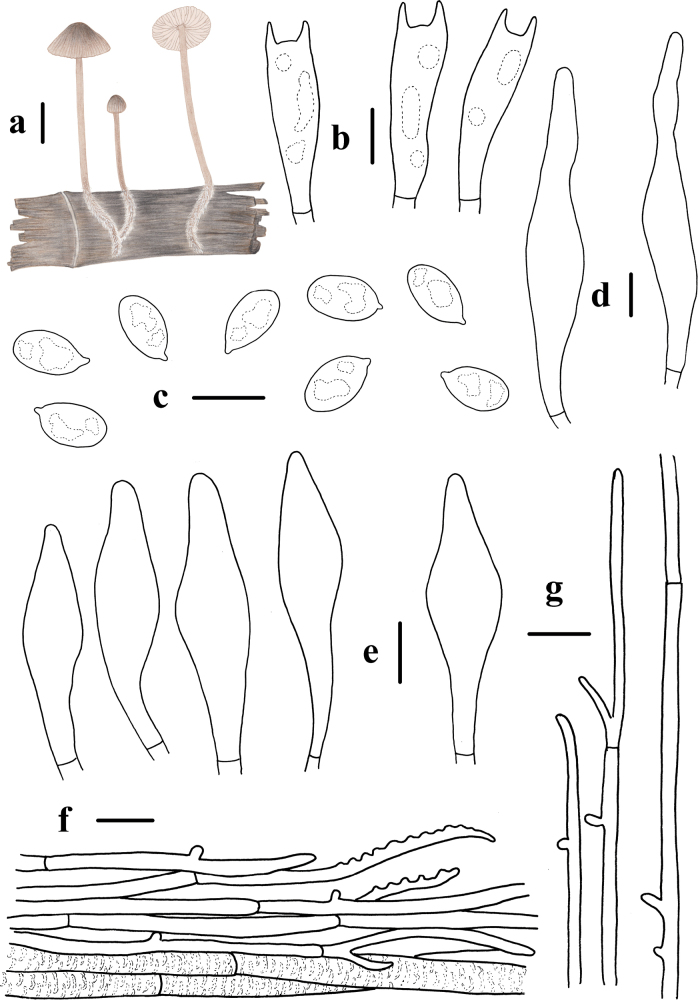
Morphological features of *Mycenaroseolamellata***a** basidiomata **b** basidia **c** basidiospores **d** pleurocystidia **e** cheilocystidia **f** pileipellis **g** hyphae of stipitipellis. Scale bars: 1 cm (**a**); 10 μm (**b–g**).

##### Habitat.

Gregarious on decayed twigs of bamboo or woody debris of deciduous trees.

##### Known distribution.

Ningxiang City, Hunan Province.

##### Additional materials examined.

China • Hunan Province, Ningxiang City, Biandan’ao, Lijingpu Subdistrict, 28°12'07"N, 112°32'43"E, elev. 110 m, 22 December 2023, ShengQiang Liu, *HUIF60002* (collection number NN602).

##### Notes.

*Mycenaroseolamellata* is classified into sect. Calodontes based on the smooth cheilocystidia and stipitipellis. Microscopically, *M.pura* (Pers.) P. Kumm. is the most similar to *M.roseolamellata*; however, *M.pura* is distinguished from *M.roseolamellata* by its purple pileus with pinkish or brown tints, lamellae interveined with age, the presence of clamp connections in all tissues, and the absence of a root-like, pruinose stipe (Robich 2003; Aronsen and Læssøe 2016). *Mycenarosea* Gramberg is somewhat similar to *M.roseolamellata*; they have pink lamellae, smooth cheilocystidia, and pleurocystidia. *Mycenarosea* can be distinguished from *M.roseolamellata* by having a pink pileus with a dull yellow center, a white or pink stipe, and the presence of clamp connections in all tissues (Robich 2003; Aronsen and Læssøe 2016). *Mycenaroseolamellata* is very different from any species of sect. Calodontes, owing to its brown pileus. *Mycenagalericulata* (Scop.) Gray shares some similarities with *M.roseolamellata* in terms of pileus color, but *M.galericulata* is differentiated by the presence of non-smooth cheilocystidia, pileipellis, and stipitipellis (Maas Geesteranus 1992a, 1992b).

## ﻿Discussion

The combination of morphological features and phylogenetic analyses revealed the presence of 30 species in Hunan Province, including five new species. In the Maas Geesteranus classification, the new species *M.fulvomarginata*, *M.huangsangensis*, *M.subroriduliformis*, and *M.roseolamellata* are classified into sect. Rubromarginatae, sect. Polyadelphia, sect. Insignes, and sect. Calodontes, respectively (Maas Geesteranus 1980, 1992a, 1992b; Maas Geesteranus and De Meijer 1997; Robich 2003, 2016). However, we could not place the new species *M.hongfengensis* in any section at present due to its special characteristics. The taxonomy of *Mycena* is overly complex, and infrageneric classification generally relies on the reported morphology of *Mycena*, and the characteristics of some species are not consistent with the common features shared by species of the numerous sections. Therefore, we need to increase the number of related species to identify the common characteristics of those species and further improve the taxonomy of *Mycena*.

Among the 30 *Mycena* species collected in Hunan, we found *M.picta* (Fr.) Harmaja on Yuelu Mountain, Hunan. As we obtained only one basidiomata in the field, sequences of *M.picta* could not be obtained for phylogenetic analysis. However, we can confirm that the specimen is *M.picta* based on its distinct macroscopic and microscopic characteristics (Aronsen and Læssøe 2016; Bau et al. 2021). And *M.heteracantha* was also collected in Yuelu Mountain (Na and Bau 2019a). We are uncertain of the classification of *M.juniperina* Aronsen and *M.meliigena* (Berk. & Cooke) Sacc. Distinguishing the 8 related specimens we collected in Hunan was difficult because of their similar microcharacteristics and the limited number of sequences available for downloading from GenBank, although the color of their basidiomata varies. Therefore, we identified all the related specimens we collected as *M.juniperina*/*meliigena*, as we did not obtain type specimens. Additional studies are needed.

Among the phylogenetic trees, *M.hongfengensis*, *M.deeptha* Aravind. & Manim. and *M.pluteoides* T. Bau & Q. Na were grouped into clade 1. Interestingly, all species have a non-smooth pileipellis hyphae. Most species collected from Hunan are mainly concentrated in clade 2. Four new species are grouped in clade 2; all *Mycena* species in this clade have a stipe or a stipe base that is covered with white fibrils.

The distinct topography, climate, and flora of Hunan Province have produced abundant and distinctive *Mycena* specimens. We conducted a comparative analysis of the geographical compositions of the 30 *Mycena* species we collected and preliminarily divided the distribution of the genus *Mycena* in Hunan Province into the following four types (Table 1). The analysis of the floristic components of the species reveals that *Mycena* in Hunan Province is cosmopolitan, exhibiting tropical-subtropical floristic characteristics as well as a certain proportion of northern temperate characteristics, indicating that *Mycena* species in Hunan possess diverse and transitional features.

Although *Mycena* was widely distributed in the world, the earliest and most detailed research was conducted in Europe and North America (Singer and Digilio 1951; Robich 2003, 2016). The morphological and microscopic characteristics of the *Mycena* species collected in Hunan Province are basically consistent with those of materials from Europe. However, the sizes of the basidiomata, basidiospores, basidia, cheilocystidia, and caulocystidia occasionally vary subtly. This may be due to the geographical location and environmental changes in the species at that time. From a geographical point of view, the *Mycena* species we collected show some differences compared with those documented in monographs from Europe and North America. Free lamellae are important for diagnosing these species in *Mycena*. We found that *Mycena* with free lamellae are more commonly collected in Hunan Province than in temperate regions in China; for example, *M.deeptha* and *M.pluteoides*, belonging to sect. Exornatae Maas Geest, are widely distributed in the western and southern areas of Hunan. And members of the sect. Exornatae are most commonly found in subtropical regions of Asia (Desjardin et al. 2010; Aravindakshan et al. 2012; Aravindakshan and Manimohan 2013a; Bau et al. 2021). These findings also indirectly suggest that sect. Exornatae has a certain preference for geographical location. Our research may contribute to the exploration of the origin and evolution of *Mycena*.

### ﻿Key to the known species of *Mycena* in Hunan Province

**Table:** 

1	Stipe arising from a basal disc	**2**
–	Stipe not arising from a basal disc	**3**
2	Dermatocysts present in the pileipellis and stipitipellis	***M.hongfengensis***
–	Dermatocysts absent in the pileipellis and stipitipellis	**4**
3	Fresh and young basidiomata exude colored fluid when damaged	***M.haematopus***
–	Basidiomata do not exude colored fluid when damaged	**7**
4	Pileus glabrous, viscid, white, with a pale brown center, depressed at the center	**5**
–	Pileus dry, pubescent, pure white, not depressed at center	**6**
5	Cheilocystidia vesiculose, smooth	***M.deeptha***
–	Cheilocystidia densely covered with projections	***M.pluteoides***
6	Basidiomata growing on *Castanea* burs, pileus slightly pubescent	***M.castaneicola***
–	Basidiomata growing on dead wood or humus layer, pileus with bran-like covering	***M.heteracantha***
7	Lamellae not white, or occasionally white when young	**8**
–	Lamellae white	**9**
8	Lamellae faces pink, occasionally white when young, cheilocystidia hyaline	***M.roseolamellata***
–	Lamellae faces orange-yellow, cheilocystidia with yellow contents	***M.leaiana***
9	Cheilocystidia smooth	**10**
–	Cheilocystidia with simple to branched excrescences	**21**
10	Lamellae faces not concolorous with the sides	**11**
–	Lamellae faces concolorous with the sides	**12**
11	Lamellae edges light yellow	***M.citrinomarginata***
–	Lamellae edges light brown to yellowish-brown	***M.fulvomarginata***
12	Hyphae of the pileipellis smooth	**13**
–	Hyphae of the pileipellis diverticulate	**16**
13	Pileus brown	***M.algeriensis***
–	Pileus violet	**14**
14	Pleurocystidia absent	**15**
–	Pleurocystidia present	***M.pura***
15	Pileipellis not gelatinized	***M.yuezhuoi***
–	Pileipellis gelatinized	***M.pearsoniana***
16	Lamellae adnate or adnexed	**17**
–	Lamellae decurrent	**18**
17	Pileus grey brown	**19**
–	Pilues white	**20**
18	Cheilocystidia thick-walled	***M.subpiligera***
–	Cheilocystidia thin-walled	***M.digitifurcata***
19	Hyphae of the stipitipellis smooth, caulocystidia present	***M.leptocephala***
–	Hyphae of the stipitipellis covered with warty or diverticulae, caulocystidia absent	***M.abramsii***
20	Pileipellis and stipitipellis gelatinized	***M.laevigata***
–	Pileipellis and stipitipellis not gelatinized	***M.adnexa***
21	Basidiomata sticky	***M.subroriduliformis***
–	Basidiomata dry	**22**
22	Pileus with yellow tone	**23**
–	Pileus without yellow tone	**24**
23	Pileus bucket-shaped, lamellae broader than the length	***M.picta***
–	Pileus hemispherical, parabolical to convex, lamellae broad	***M.meliigena* / *M.juniperina***
24	Basidiospores globose	***M.corynephora***
–	Basidiospores broadly ellipsoid to ellipsoid	**25**
25	Pileus and lamella with red spots when old	***M.maculata***
–	Pileus and lamella without red spots when old	**26**
26	Rhizomorphs present	***M.chlorocyanea***
–	Rhizomorphs absent	**27**
27	Clamp connections absent in all tissues	***M.galericulata***
–	Clamp connections present in all tissues	**28**
28	Acanthocysts present, acanthocysts of two types, pyriform to vesicular	***M.bicystidiata***
–	Acanthocysts absent	**29**
29	Pileus and stipe pruninose, iodoform when dry	***M.filopes***
–	Pileus and stipe glabrous, odor indistinctive	***M.huangsangensis***

## Supplementary Material

XML Treatment for Mycena huangsangensis

XML Treatment for Mycena fulvomarginata

XML Treatment for Mycena hongfengensis

XML Treatment for Mycena subroriduliformis

XML Treatment for Mycena roseolamellata

## ﻿Additional information

### Author ORCIDs

Ying Xin Xiao https://orcid.org/0009-0008-7041-503X

Li Na Liu https://orcid.org/0000-0003-3241-4016

Zhu Ming Tan https://orcid.org/0000-0001-6696-1179

Ai Rong Shen https://orcid.org/0000-0003-4543-0250

Bao Ming Shen https://orcid.org/0000-0003-0110-3370

Yun Tan https://orcid.org/0000-0002-8129-0163

Sai Nan Li https://orcid.org/0000-0002-8818-446X

Zhu Xiang Liu https://orcid.org/0000-0001-6014-3554

### Data availability

All of the data that support the findings of this study are available in the main text.
